# Morphological and histological description of the midgut caeca in true crabs (Malacostraca: Decapoda: Brachyura): origin, development and potential role

**DOI:** 10.1186/s40850-022-00108-x

**Published:** 2022-02-04

**Authors:** Diego Castejón, Guiomar Rotllant, Javier Alba-Tercedor, Enric Ribes, Mercè Durfort, Guillermo Guerao

**Affiliations:** 1 Centro de Maricultura da Calheta, Avenida D. Manuel I 7, 9370-135 Calheta, Madeira Portugal; 2 Centre d’ Aqüicultura, IRTA, Ctra. del Poble Nou Km 5.5, 43540 Sant Carles de la Ràpita Tarragona, Spain; 3 Institut de Ciències del Mar, CSIC, Passeig Marítim de la Barceloneta 37‑49, 08003 Barcelona, Spain; 4 Departamento de Zoología, Facultad de Ciencias, Universidad de Granada, Campus de Fuentenueva, Av. de Fuente Nueva s/n, 18071 Granada, Spain; 5 Unitat de Biologia Cel·lular, Departament de Biologia Cel·lular, Fisiologia i Immunologia, Facultat de Biologia, Universitat de Barcelona, Diagonal 645, 08028 Barcelona, Spain; 6 Independent Researcher, Barcelona, Spain

**Keywords:** Larvae, Anterior midgut caeca, Posterior midgut caecum, Macroapocrine secretion, Microapocrine secretion, Endoderm germ layer

## Abstract

**Background:**

The decapods are a major group of crustaceans that includes shrimps, prawns, crayfishes, lobsters, and crabs. Several studies focused on the study of the digestive system of the decapods, constituted by the oesophagus, stomach, midgut tract, midgut gland, and hindgut. Nevertheless, in the midgut tract there are associated a set of organs called “midgut caeca”, which are among the most controversial and less studied digestive organs of this group. This work used the common spider crab *Maja brachydactyla* Balss, 1922 as a model to resolve the origin, development, and potential role of the midgut caeca. Such organs were studied in the larvae (zoea I, zoea II, megalopa), first juveniles, and adult phases, being employed traditional and modern techniques: dissection, micro-computed tomography (Micro-CT), and light and electron microscopical analyses (TEM and SEM).

**Results:**

The common spider crab has a pair of anterior midgut caeca and a single posterior caecum that originate from the endoderm germ layer: they develop from the midgut tract, and their epithelium is composed by secretory cells while lacking a cuticle lining. The midgut caeca are small buds in the newly hatched larvae, enlarge linearly during the larval development, and then continue growing until became elongated and coiled blind-tubules in adults. The adult midgut caeca are internally folded to increase their inner surface. The electron microscopy observations showed that the midgut caeca are highly active organs with important macroapocrine and microapocrine secretory activity. Our results suggest that the role of the caeca might be related to the digestive enzyme secretion. The secretory activity should increase as the animal grows in size.

**Conclusion:**

The present study resolves the embryonic origin of the midgut caeca (endoderm derived organs), development (general lengthening starting from small buds), and role (active secretory organs). The secretory activity of the midgut caeca should be incorporated in the current models of the digestive physiology in different decapod taxa.

**Supplementary Information:**

The online version contains supplementary material available at 10.1186/s40850-022-00108-x.

## Background

The decapods comprises a major malacostracean (Crustacea sensu *lato*) group with over 15,000–17,000 recognized species distributed in around 2800 genera [1–3]. The decapods includes several economically important species, such as crabs, crayfishes, lobsters, prawns and shrimps, as well other lesser groups such as the ghost shrimps and the deep-sea polychelids [1–5]. The monophyly of the decapods is supported by several studies [3, 6–8], and the true crabs (Brachyura Latreille, 1802) are probably one of the most diverse and ubiquitous decapod taxa with over 7250 species in 93 families [9, 10]. These crabs can be found in marine, estuarine, freshwater, and terrestrial environments, from mountainous landscapes to the deep-sea floor [9].

The brachyurans are a relatively well studied group of decapods that have been used as a model to study the crustacean digestive system since the astonishing atlas elaborated by Milne-Edward in the early nineteenth-century [11]. Several studies have been published since then [12–20], and notable reviews of comparative anatomy with other crustacean taxa are available [21–27]. In general, the morphology of the digestive system of the brachyurans is shared with other decapods [18, 24, 25, 28]: the mouth connects with a short muscular oesophagus lined by a cuticle, it is followed by a stomach with several calcified pieces (named ossicles) and a gastric mill composed by cuticular teeth. The terminal portion of the stomach connects with the voluminous midgut gland (a.k.a. hepatopancreas), responsible of the digestion and nutrient absorption; and the midgut tract, responsible of the secretion of the perithrophic membrane. The terminal portion of the midgut tract continues with the hindgut tract, which is involved in the excretion of solid residuals and osmoregulation.

The midgut tract is associated with other set of organs that are called jointly as midgut caeca, which are probably the most neglected and controversial structures of the digestive system of the decapods. The midgut caeca are traditionally known as blind tubes which morphology is highly different among taxa: from very short finger-like projections in crayfishes [29, 30], to very long and coiled tubules in brachyuran and anomuran crabs [31]. The midgut caeca have been subject of debate since their description two centuries ago [11]. In this sense, Smith [31] reported several misconceptions at the time and postulated that crabs (brachyurans) have three caeca: a pair of “anterior caeca” (AC), raising from the stomach-midgut junction and projected forward; and a single “posterior caecum” (PC), raising from the midgut-hindgut junction and projected backward.

The controversy regarding the midgut caeca of the decapods includes their origin, development, and role. The origin of the midgut caeca has been traditionally associated either with the ectoderm layer (like the stomach and hindgut) or with the endoderm layer (like the midgut tract and the midgut gland). RI Smith [31] stated that such controversy leaded to historical denominations such as “pyloric caeca”, “hindgut caecum” or “rectal caecum”. Currently, the most accepted proposal states that the caeca arise from the midgut tract, being currently denominated as “midgut caeca” [27, 31]. The development of the caeca have been studied in a few decapod species showing a high degree of variability: in king crabs the caeca enlarge during the larval development [32], in penaeid shrimps the posterior caecum first appears as a small outpocketing of the midgut tract which develops into a cauliflower shaped structure in adults [33], while in clawed lobsters the caeca degenerate during development [29, 30]. At this regard, the development of the midgut caeca in true crabs is poorly known and little literature is available thus far [19, 34, 35]. Finally, the role of the midgut caeca remains in the mystery, albeit some suggestions have been realized. CM Yonge [36] cautiously proposed a role related with the nutrient absorption, Holliday et al. [37] proposed a role related with the regulation of the body fluids along the moulting cycle, and Smith [31] pointed that the caeca are “little understood functionally”.

This study is focused on the midgut caeca of the common spider crab *Maja brachydactyla* Balss, 1922 as model of eubrachyuran species. This marine brachyuran is distributed in the eastern Atlantic coast from the British Isles to the Sahara and SW Mediterranean Sea [38, 39], and support several fisheries along several European coasts (Spain, Portugal, France, and Ireland) [40, 41]. Genetic studies support the Atlantic *M. brachydactyla* as a different species from the Mediterranean *M. squinado* [38, 39, 42, 43]. This species have the advantages to be easy to breed in captivity, having a short larval development that can be completed in around 2 weeks without special requirements [44, 45]. Larval development consists in two free swimming zoeal stages (zoea I and zoea II), and a single pelagic-benthic megalopa stage, which metamorphoses into a benthic juvenile [46, 47]. The common spider crab has been useful to study the ontogeny of the digestive organs in brachyurans, e.g. oesophagus [48], stomach [49, 50], midgut gland [20], and hindgut tract [51].

The objectives of the present study were to insight into the three major questions regarding the midgut caeca in brachyurans: origin, development, and potential role. For this purpose, the anterior and posterior midgut caeca of the common spider crab were studied in larvae (zoea I, zoea II and megalopa stages), first juvenile, and adult phases. The techniques employed were a combination of classical and modern approaches: dissection, micro-computed tomography (Micro-CT), and light and electron microscopical analyses (TEM and SEM). Thus, has been study the location, development, cellular and sub-cellular organization, and activity of the midgut caeca. Finally the potential role of the midgut caeca is discussed.

## Results

### Morphology and location of the midgut caeca

**Fig. 1:**
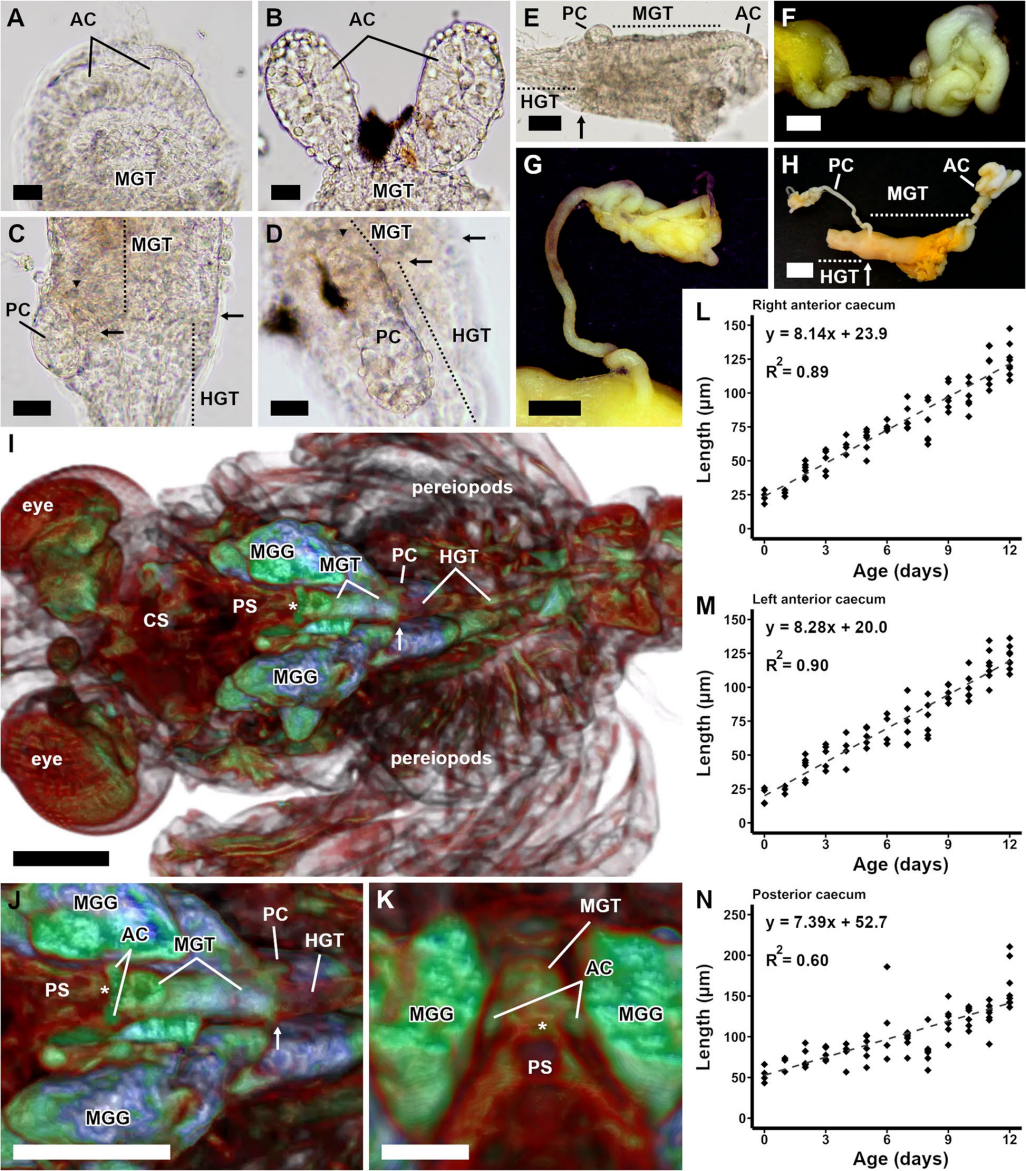
*Maja brachydactyla*. Gross morphology and development of the midgut caeca. Anterior caeca (**A–B**): newly hatched zoea I (**A**) and juvenile 12 days post-hatching (**B**). Posterior caecum (**C–D**): newly hatched zoea I (**C**) and juvenile 12 days post-hatching (**D**). Isolated midgut tract and associated caeca, newly hatched zoea I (E). Midgut caeca, adult (**F–G**): anterior caeca (**F**), and posterior caecum (**G**). Isolated midgut tract and associated caeca, adult (**H**). Midgut tract and associated caeca, megalopa 6 days post-hatching, micro-CT rendered image reconstructions, dorsal section (**I–J**). Anterior caeca, megalopa 6 days post-hatching, micro-CT rendered image reconstruction, transversal section (**K**). Length growth of the midgut caeca from hatching to the first juvenile stage (**L–N**): right anterior caecum (**L**), left anterior caecum (**M**), and posterior caecum (**N**). Scale bars (μm): 25 (**A–D**), 50 (**E**), 100 (**K**), 250 (**I–J**); (mm): 2 (**F–G**), 10 (**H**). Abbreviations: AC, anterior caeca; arrow, midgut-hindgut junction; arrowhead, junction between the posterior midgut caecum and the midgut tract; asterisk, stomach-midgut junction; CS, cardiac stomach; HGT, hindgut tract; MGG, midgut gland; MGT, midgut tract; PC, posterior caecum; PS, pyloric stomach

The common spider crab *M. brachydactyla* has a pair of anterior midgut caeca (AC), and a single posterior midgut caecum (PC) since hatching (Fig. 1). The AC of the newly hatched larvae are paired buds arising dorsally from the anterior tip of the midgut tract in the stomach-midgut junction (Fig. 1A, E). On the other hand, the PC of the newly hatched larvae is a single short blind-tube that arises from the latero-dorsal side of the midgut tract in the midgut-hindgut junction (Fig. 1C, E). All the midgut caeca enlarged during the larval development following a linear model (Fig. 1A–D, L–N). The micro-CT reconstruction of the megalopa larva showed that the AC are projected forward from the anterior tip of the midgut tract to fit between the lateral walls of the pyloric stomach and the midgut gland (Fig. 1I–K; Supl. video 1), while the PC projects backward from the latero-dorsal side of the midgut tract on the midgut-hindgut junction (Fig. 1I–J; Supl. video 1). The micro-CT also showed that all the midgut caeca have X-ray opacity similar to the midgut tract rather than to the stomach or the hindgut tract (Fig. 1I–K; Supl. video 1). The adult specimens have a pair of AC and a single PC arising from the same sites than the described in the larvae and juveniles (Fig. 1F–H). At difference from previous stages, the AC and PC of the adults are very long tubules coiled around themselves (Fig. 1F–H). In adults, the AC start to coil after arising from the anterior tip of the midgut tract, the coiled portion represents the longer length of the AC and it is attached to the lateral walls of the pyloric stomach (Fig. 1F). Meanwhile, the thinner PC attaches to the hindgut tract after arising from the posterior end of the midgut tract, the linear portion is very short and it is followed by a long coiled portion (Fig. 1G).

### Tissue organization of the midgut caeca

**Fig. 2:**
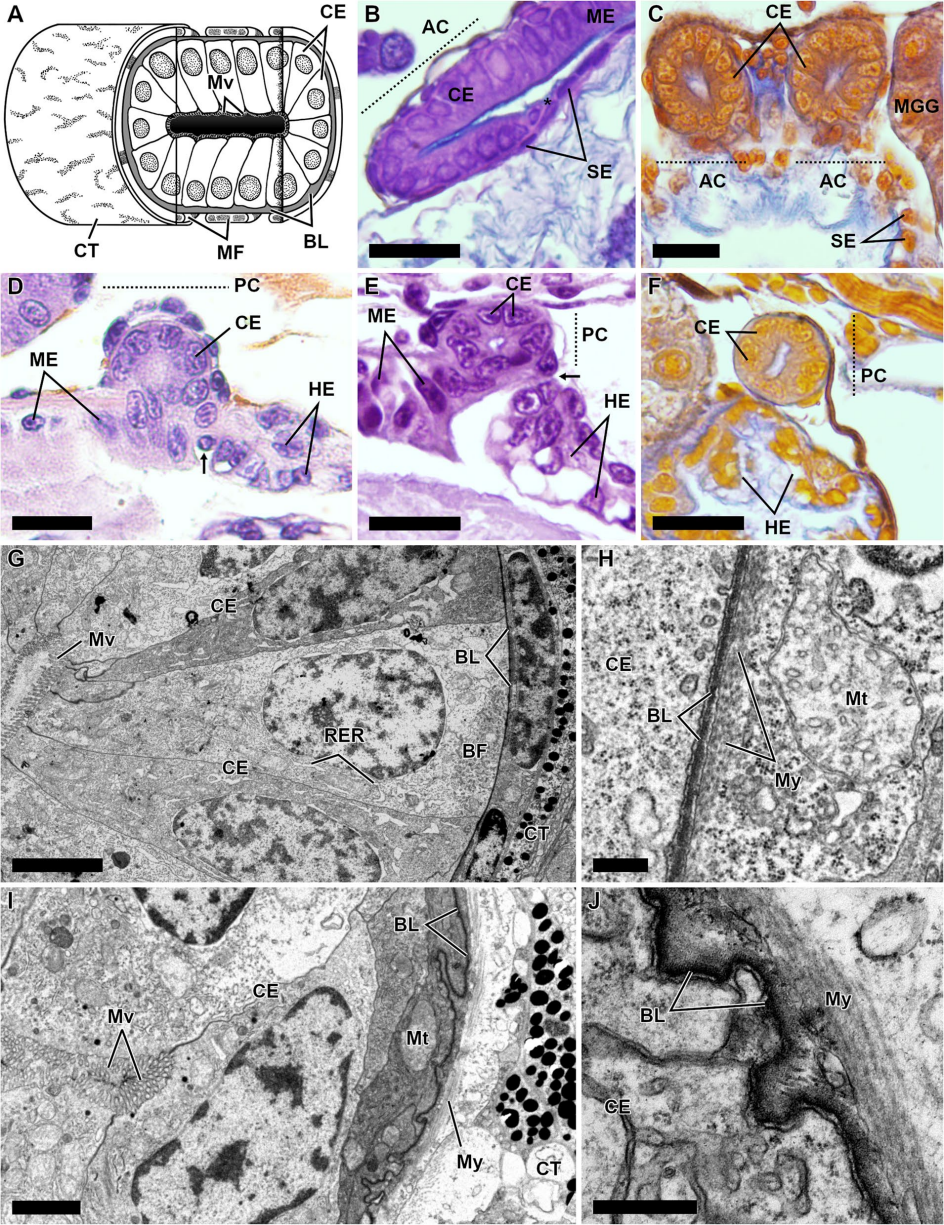
*Maja brachydactyla*. Larval stages (zoea I, zoea II, and megalopa). Tissue organization of the midgut caeca. General diagram of the larval midgut caeca (**A**). Anterior midgut caeca (**B–C**): zoea II 6 days post-hatching, PAS-Alcian Blue contrasted with Haematoxylin, longitudinal cross section (**B**); and megalopa 6 days post-hatching, Mallory’s Trichrome, transversal cross section (**C**). Transition between the posterior midgut caecum and the midgut tract, Haematoxylin-Eosin, longitudinal cross section of the specimen (**D–E**): megalopa 6 days post–hatching (**D**), and newly hatched zoea I (**E**). Posterior midgut caecum, zoea II 6 post-hatching old, Mallory’s Trichrome, transversal cross section of the specimen (**F**). Anterior midgut caeca, megalopa 10 days post-hatching, TEM (**G–H**): general view (**G**), and detail of the myofibrils-like filaments (**H**). Posterior midgut caeca, megalopa 10 days post-hatching, TEM (**I–J**): general view (**I**), and detail of the myofibrils-like filaments (**J**). Scale bars (nm): 500 (**H, J**); (μm): 2 (**I**), 5 (**G**), 20 (**B–F**). Abbreviations: AC, anterior midgut caecum; arrow, midgut- hindgut junction; asterisk, midgut-stomach junction; BF, basal folds; BL, basal lamina; CE, midgut caeca epithelium; CT, connective tissue; HE, hindgut tract epithelium; ME, midgut tract epithelium; MF, muscle fibres; MGG, midgut gland; Mt, mitochondria; Mv, microvilli; My, myofibrils-like filaments; RER, rough endoplasmic reticulum; SE, stomach epithelium; PC, posterior midgut caecum

The midgut caeca (AC and PC) during the early life (larval and first juvenile stages) show an organization composed by four structures: epithelium, basal lamina, muscle fibres, and connective (Fig. 2). The lumen is enclosed by a simple, cuboidal to short columnar epithelium crowned by short microvilli (Fig. 2A–G, I). The light microscopy observations confirmed that AC and PC develop from the midgut tract: the AC extend from the anterior tip of the midgut tract just after the stomach-midgut junction (Fig. 2B), and the PC extends from the posterior end of the midgut tract just before the midgut-hindgut junction (Fig. 2D–E). The transition between the epithelia of the midgut tract and the epithelia of the midgut caeca is smooth (Fig. 2B, D). The epithelium is supported on a thin and electron-dense basal lamina (Figs. 2G–J; 4C, E, H). The basal lamina is followed by a single, discontinuous layer of muscle fibres with myofibrils-like filaments (Figs. 2H–J; 4C). The muscle fibres look like embedded in another single cell layer resembling a connective, in this layer has been found cells rich in electron-dense granules (Fig. 2G, I).

**Fig. 3:**
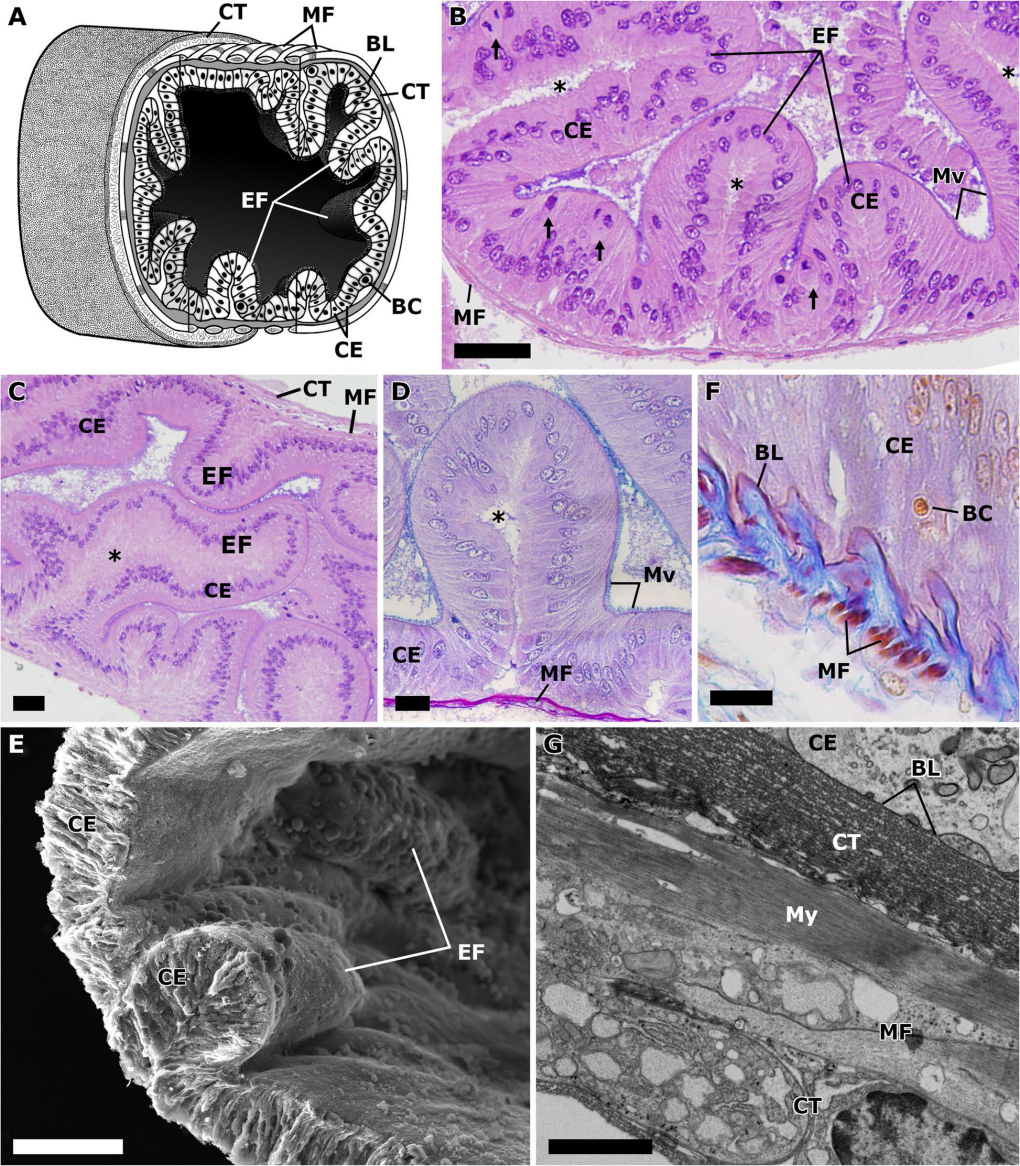
*Maja brachydactyla*. Adults. Tissue organization of the midgut caeca. General diagram of the adult midgut caeca (**A**). Anterior midgut caeca, general view, Haematoxylin-Eosin (**B**). Posterior midgut caecum, general view, Haematoxylin-Eosin (**C**). Anterior midgut caeca, detailed view of a caeca fold, PAS-Alcian Blue contrasted with Haematoxylin (**D**). Anterior midgut caeca, epithelial folds, SEM (**E**). Anterior midgut caeca, detailed view of the muscle fibres and connective, Mallory’s Trichrome (**F**). Posterior midgut caecum, detailed view of the muscle fibres and connective, TEM (**G**). Scale bars (μm): 2 (**G**), 20 (**D, F**), 50 (**B–C**), 100 (**E**). Abbreviations: arrow, cellular division; asterisk, inner layer of connective tissue; BC, basal cells; BL, basal lamina; CE, midgut caeca epithelium; CT, external layer of connective tissue; EF, epithelial folds; MF, muscle fibres; Mv; microvilli; My, myofibrils

The midgut caeca (AC and PC) of the adults have an organization similar to the one described in the larval stages. The same structures were observed with a bigger size: epithelium, basal lamina, muscle fibres, and connective (Fig. 3). The lumen is enclosed by a simple, tall columnar epithelium crowned by microvilli (Fig. 3A–E). The epithelium of the adults is internally folded (Figs. 3B–D, E; 7D–E). The folds are epithelial elevations with an elliptical and elongated shape, oriented from parallel to perpendicularly with the caeca length (Figs. 3E; 7D–E). The epithelium is supported on the basal lamina (Fig. 3G). The caeca are limited by a circular perimeter in which are located the thin muscle fibres, which distribution is dominantly circular (Fig. 3B, D, F, G). The muscle fibres are attached or embedded in a thin layer of connective (Fig. 3G).

### The epithelium of the midgut caeca

**Fig. 4:**
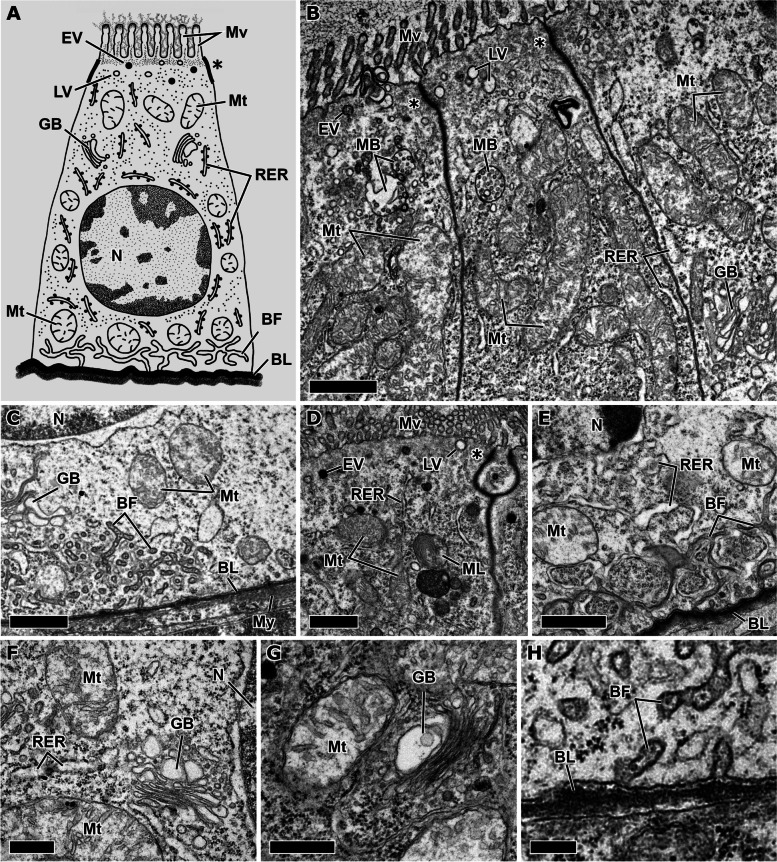
*Maja brachydactyla*. Megalopae 10 days post–hatching. Epithelium of the midgut caeca. TEM. General diagram (**A**). Anterior midgut caeca (**B–C**): supranuclear region (**B**), and infranuclear region (**C**). Posterior midgut caecum (**D–E**): supranuclear region (**D**), and infranuclear region (**E**). Detailed view of the mitochondria and Golgi bodies (**F–G)**: anterior midgut caeca (**F**), and posterior midgut caecum (**G**). Anterior midgut caeca, detailed view of the basal folds of the epithelial cells (**H**). Scale bars (nm): 200 (**H**), 500 (**F–G**); (μm): 1 (**B–E**). Abbreviations: asterisk, cell-to-cell junction; BF, basal folds; BL, basal lamina; EV, electron-dense vesicles (cytoplasm); GB, Golgi bodies; LV, lucent vesicles (cytoplasm); MB, multivesicular body; ML, multilamellar body; Mt, mitochondria; Mv, microvilli; My, myofibrils; N, nucleus; RER, rough endoplasmic reticulum

The epithelium of the AC and the PC is similar during the larval stages (Fig. 4). The electron microscopy showed that the epithelial cells have a polarized organization. The apical membrane forms very short and slightly undulated microvilli with an electron-dense apex (Figs. 4B, D; 7A–C). The microvilli are in direct contact with the lumen of the caeca (Figs. 4B, D; 7A–C). Cell–to–cell junctions were observed on the lateral membranes of the cell apex (Figs. 4B, D; 7A–B). The lateral and basal membranes of the epithelial cells are generally smooth (Fig. 4B–E). Basal folds extending in tubular structures occurred on the basal membrane (Fig. 4C, E, H). The supranuclear region contains some lucent and electron-dense vesicles in the cell apex, as well multilamellar and multivesicular bodies (Fig. 4B, D). Below the vesicles, the cytoplasm is rich in mitochondria (Fig. 4B, D), followed by numerous Golgi complexes and elongated cisternae of rough endoplasmic reticulum (Fig. 4B, D, F–G). The entire cytoplasm is very rich in ribosomes (Fig. 4).

**Fig. 5:**
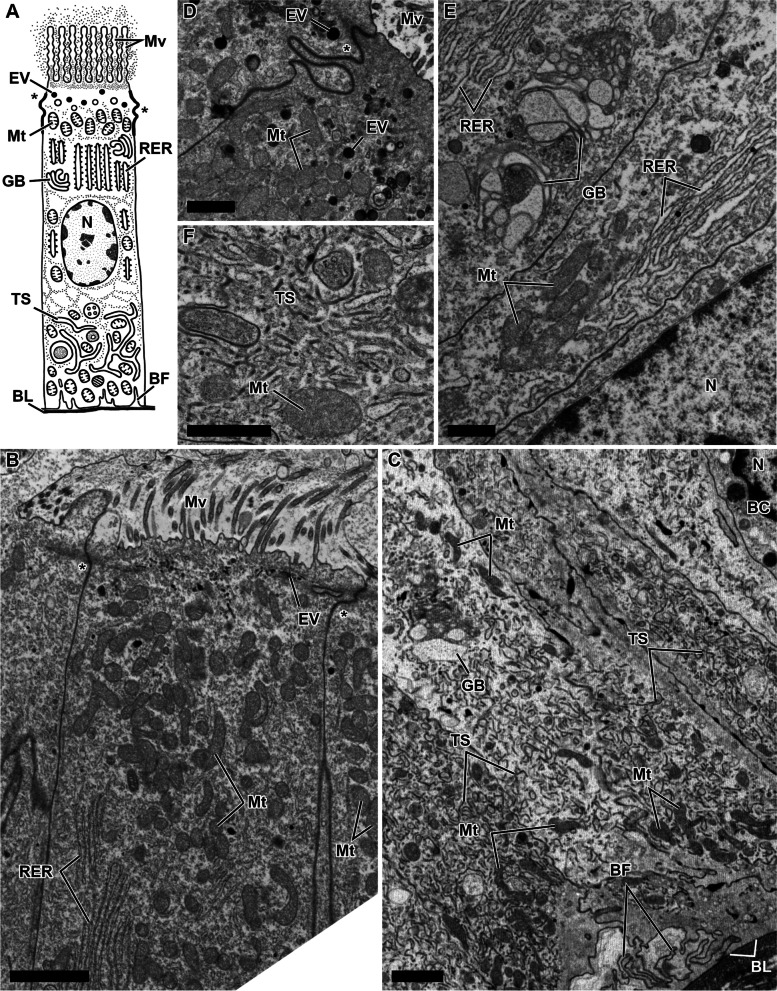
*Maja brachydactyla*. Adults. Epithelium of the midgut caeca: epithelial cells. Epithelial cell, general diagram (**A**). Anterior midgut caecum, epithelial cell (**B–C**): supranuclear region (**B**), and infranuclear region (**C**). Posterior midgut caeca, epithelial cell, close view of different structures (**D–F**): cell-to-cell junction (**D**), rough endoplasmic reticulum, mitochondria, and Golgi complexes (**E**), and smooth endoplasmic reticulum, and mitochondria (**F**). Scale bars (nm): 500 (**F**); (μm): 1 (**D–E**), 2 (**B–C**). Abbreviations: asterisk, cell-to-cell junction; BC, basal cell; BF, basal folds; BL, basal lamina; EV, electron dense-vesicles (cytoplasm); GB, Golgi bodies; Mt, mitochondria; Mv, microvilli; N, nucleus; RER, rough endoplasmic reticulum; TS, tubular structures

**Fig. 6:**
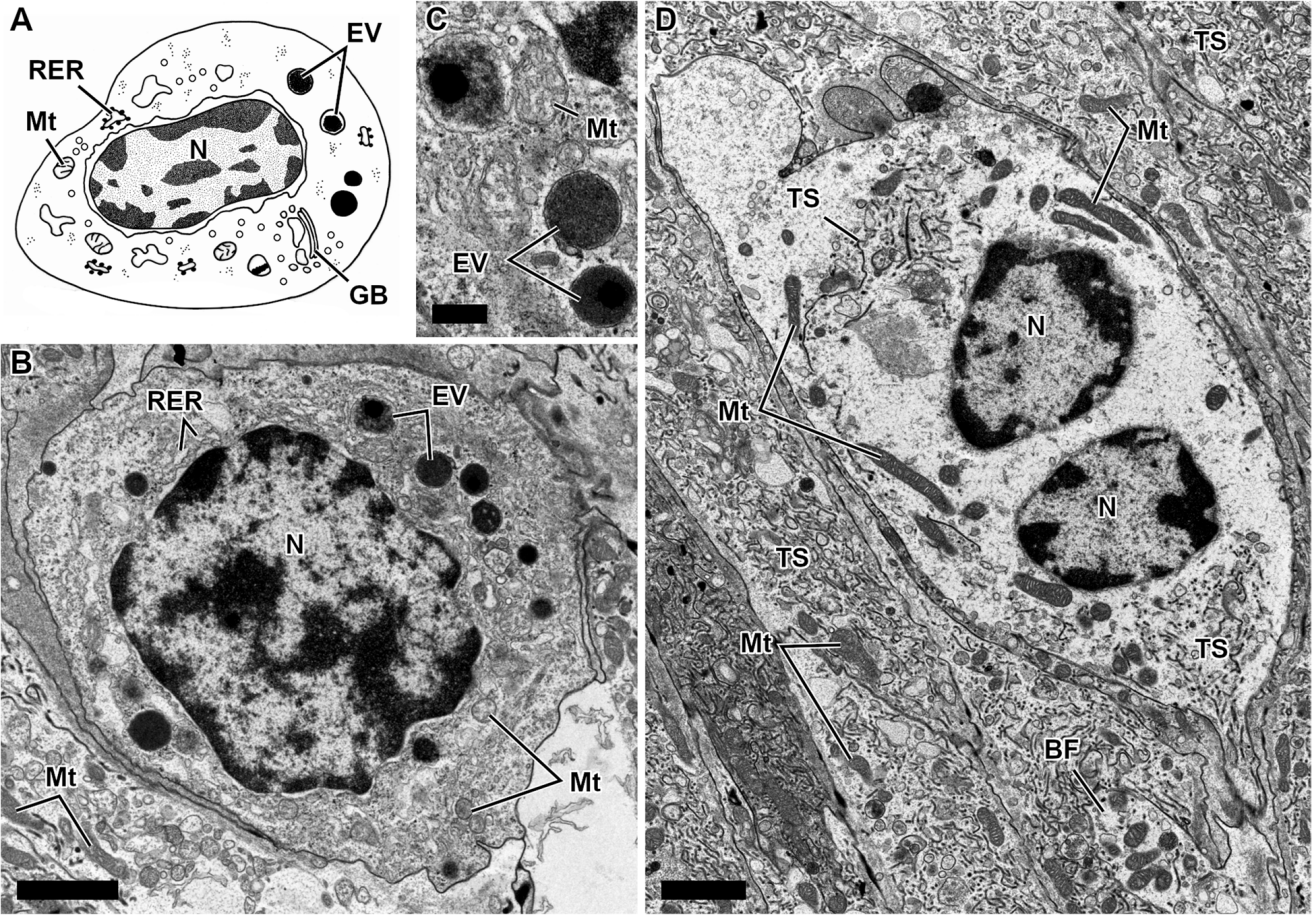
*Maja brachydactyla*. Adults. Epithelium of the midgut caeca: basal cells and cell division. Basal cell, general diagram (**A**). Anterior midgut caecum, basal cell, general view (**B**). Anterior midgut caecum, basal cell, close view of the electron-dense vesicles and mitochondria (**C**). Anterior midgut caecum, cellular division (**D**). Scale bars (nm): 500 (**C**); (μm): 2 (**B, D**). Abbreviations: BF, basal folds; EV, electron dense-vesicles (cytoplasm); GB, Golgi bodies; Mt, mitochondria; N, nucleus; RER, rough endoplasmic reticulum; TS, tubular structures

**Fig. 7:**
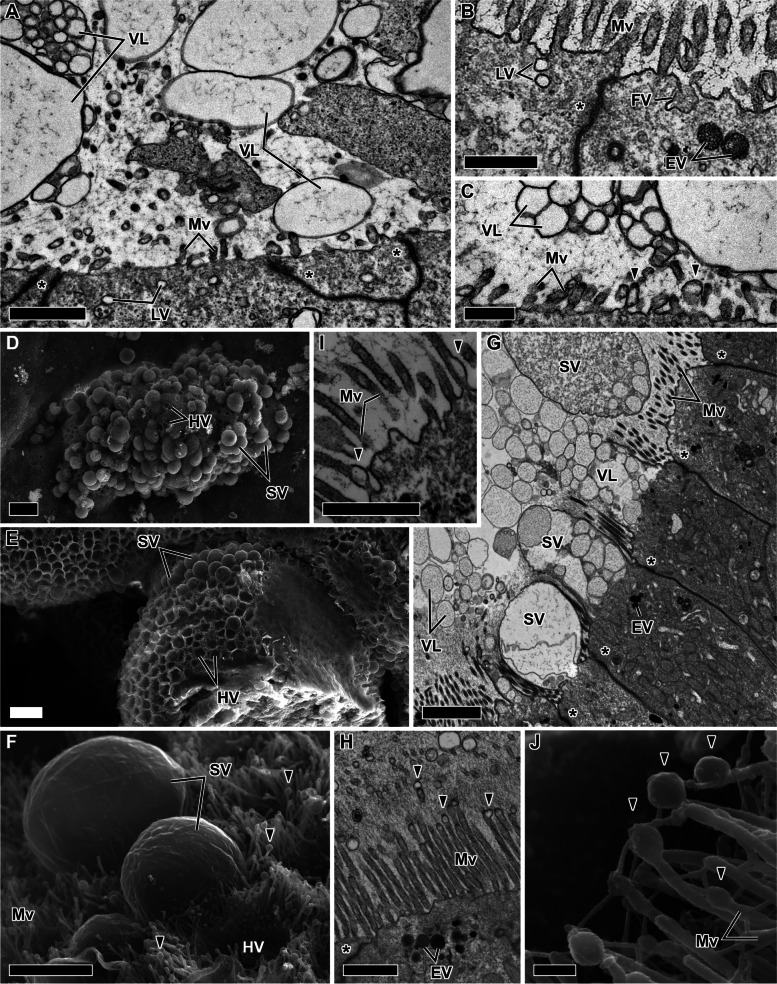
*Maja brachydactyla*. Secretory activity of the midgut caeca. Anterior midgut caeca, zoea II 6 days post-hatching, cell apex and lumen, TEM (**A**). Anterior midgut caeca, megalopa 10 days post-hatching, fusion between vesicles and apical cell membrane, TEM (**B**). Anterior midgut caeca, zoea II 6 days post-hatching, microvillus distended, TEM (**C**). Anterior midgut caeca, adult, epithelial fold and brush border surface, SEM (**D**). Posterior midgut caecum, adult, epithelial folds and brush border surface, SEM (**E**). Posterior midgut caecum, adult, detailed view of macro-apocrine secretion (secretory vesicles), SEM (**F**). Anterior midgut caeca, adult, detailed view of macro-apocrine secretion including cell apex and lumen, TEM (**G**). Anterior midgut caeca, adult, microvillus distended, TEM (**H–I**). Posterior midgut caecum, adult, microapocrine secretion (detailed view of the tiny secretion vesicles of the microvilli), SEM (**J**). Scale bars (nm): 500 (**B–C, J**); (μm): 1 (**A, H–I**), 2 (**G**), 5 (**F**), 20 (**D–E**). Abbreviations: arrowhead, vesicle-like distension of the microvillus; asterisk, cell-to-cell junction; EV, electron-dense vesicles (cytoplasm); FV, potential fusion between vesicle and apical cell membrane; HV, holes left after the release of the vesicles; LV, lucent vesicles (cytoplasm); Mv, microvilli; SV, secretory vesicles; VL, vesicle of the lumen

In adults, the AC and the PC have a similar epithelium in which were identified two cell types: epithelial cells occupying the entirety of the epithelium and forming folds (Figs. 3B–F; 5), and scarce basal cells located within the epithelial cells (Figs. 3F; 6B). Regarding the epithelial cells, the apical membrane forms large and undulated microvilli with an electron-dense apex (Figs. 5B; 7F–H). The lateral membranes are generally smooth and straight (Fig. 5B, E), but two exceptions: the cell apex which shows cell-to-cell junctions extended up to 4–5 μm and occasionally folded (Figs. 5B, D; 7G), and the cell basis which can show short undulations and folds (Fig. 5C). The basal membranes can be also folded (Figs. 5C; 6D). The supranuclear region can be subdivided in three major domains. The first one is adjacent to the apical membrane and generally contains small vesicles whose content varies from lucent to electron-dense (Figs. 5B, D; 7G, H). The vesicles are followed by an area dominated by the mitochondria (Figs. 5B, D; 7G). Below the mitochondria, the cytoplasm is occupied mostly by numerous cisternae of rough endoplasmic reticulum, which are usually organized in parallel stacks aligned to the vertical axis of the cell (Fig. 5B, E). Several Golgi complexes are found usually near the cisternae of rough endoplasmic reticulum (Fig. 5E). The infranuclear region is occupied by electron-dense tubular structures surrounding mitochondria, Golgi complexes, and vesicles (Figs. 5C, F; 6D). The ribosomes are present thorough the entire cytoplasm (Figs. 5–6).

The basal cells are the other cell type of the epithelium of the midgut caeca. The basal cells are uncommon and small cells (ca. 7–10 μm diameter), which shape varies from rounded to ellipsoidal (Figs. 3F; 6B). The basal cells are found near the basal lamina and cannot reach the lumen due to their size (Fig. 3F). The basal cells do not have any clear polarization on their cell membrane or cytoplasm. The cell membranes are smooth with sparse short undulations or irregularities (Fig. 6B). The small mitochondria are located around the nucleus and the organelles. A characteristic of the basal cells is the presence of medium sized vesicles, the diameter of those vesicles varied between 0.5 and 1.0 μm and the content is highly electron-dense (Fig. 6B–C).

The cellular division have been reported on the folds of the adult epithelium (Fig. 3B). Moreover, a cell resembling an advanced telophase stage was observed by electron microscopy (Fig. 6D). Such cell showed the size of a basal cell, as well organelles with a mirrored distribution. The morphology of the mitochondria and smooth endoplasmic reticulum of this cell are similar to those of the epithelial cells (Fig. 6D).

### Activity of the midgut caeca

The midgut caeca did not show a clear activity during the larval stages. The lumen of the larval midgut caeca was filled with vesicles of variable size and surrounded by a membrane (Fig. 7A, C), but it was not possible to discern if such content was originated from the epithelial cells of the midgut caeca. In this sense, the cell apex showed a low density of vesicles, either if those were lucent or electron-dense (Figs. 4B, D; 7A–B). The vesicles can fuse with the apical membrane resembling a merocrine type of secretion, but this event was rarely observed (Fig. 7B). Some microvilli of the larval midgut caeca were distended and showed a lucent content (Fig. 7C).

By contrast, the midgut caeca of the adults (either AC or PC) showed a very high secretory activity (Figs. 3E, 7D–J). Two types of secretory activity were reported. The first one was macroapocrine secretion (Figs. 3E, 7D–G): the scanning electron microscopy observations revealed that the brush border of the epithelium is very rich in secretory vesicles, being also observed numerous hole-like formations that probably belongs to the space left by the secretory vesicles after its release (Fig. 7D–F). The transmission electron microscopy observations showed that the secretory vesicles correspond to a macroapocrine type of cellular secretion, those vesicles were formed by the expansion of the apical membrane of the epithelial cells (Fig. 7G). The content of the secretory vesicles consisted on a variable number of smaller vesicles, in turn filled by either lucent or electron-dense and granular matter (Fig. 7G). The secretory vesicles were released in two forms: 1) the whole vesicle was released, this method included its content based on smaller vesicles; and 2) the membrane of the secretory vesicles broke releasing its content based on smaller vesicles (Fig. 7G). The lumen of the adult midgut caeca is rich in vesicles of variable size and surrounded by a membrane (Fig. 7G). The second type of secretory activity was microapocrine secretion: apocrine secretion released from the microvilli of the brush border (Fig. 7F–J). Those vesicles were formed as small lucent vesicles in the microvilli with a globular shape, then these small vesicles were released into the caeca lumen (Fig. 7H–J). The microapocrine secretion from the microvilli was common to observe and simultaneous with the macroapocrine secretion from the cell apex (Fig. 7F).

## Discussion

The common spider crab *Maja brachydactyla*, from hatching to the adult phase, has a pair of anterior midgut caeca (AC) located on the anterior extreme of the midgut tract; and a single posterior caecum (PC) located on the posterior end of the midgut tract. This description coincides with the detailed anatomical illustrations of adult specimens of the genus *Maja* realized by Henri Milne-Edwards [11]. Moreover, the number of midgut caeca described in *M. brachydactyla* and their anatomical position are the same than the described in different brachyuran species (Table 1). The number of midgut caeca is generally consistent at Infraorder-level (Table 1). Similarly, certain taxa which closeness is supported by genomic studies share the same number of midgut caeca, e.g. Anomura and Brachyura, and Axiidea and Gebiidea [2, 3].

**Table 1:** Presence and number of anterior (AC) and posterior midut caeca (PC) in different crustacean taxa. In some taxa the midgut gland resembles to a caeca-like organ, such caeca are not included in this table

Species	Taxa	AC	PC	References
*Gammarus lacustris* G.O. Sars, 1863	Amphipoda	1	2	[52]
*Hyalella azteca* Saussure, 1858	Amphipoda	3	2	[53]
*Macarorchestia remyi* (Schellenberg, 1950)	Amphipoda	3	2	[54]
*Galathea squamifera* Leach, 1815	Anomura	0	0	[31]
*Lithodes maja* (Linnaeus, 1758)	Anomura	2	1	[31]
*Pagurus bernhardus (Linnaeus, 1758)*	Anomura	2	1	[55]
*Paralithodes camtschaticus* (Tilesius, 1815)	Anomura	2	1	[32]
*Homarus gammarus* (Linnaeus, 1758)	Astacidea	1	1	[56]
*Nephrops norvegicus* (Linnaeus, 1758)	Astacidea	1	1	[31]
*Axius stirhynchus* Leach, 1815	Axiidea	0	1	[57]
*Callianassa subterranea* (Montagu, 1808)	Axiidea	0	1	[57]
*Calocaris macandreae* Bell, 1853	Axiidea	0	1	[57]
*Cancer pagurus* Linnaeus, 1758	Brachyura	2	1	[31]
*Carcinus maenas* (Linnaeus, 1758)	Brachyura	2	1	[31]
*Hyas araneus* (Linnaeus, 1758)	Brachyura	2	1	[31]
*Liocarcinus depurator* (Linnaeus, 1758)	Brachyura	2	1	[31]
*Maja brachydactyla* Balss, 1922	Brachyura	2	1	Present study
*Menippe rumphii* (Fabricius, 1798)	Brachyura	2	1	[58]
*Metacarcinus magister* Dana, 1852	Brachyura	2	1	[59]
*Oziothelphusa senex* (Fabricius, 1798)	Brachyura	2	1	[31]
*Parathelphusa convexa* De Man, 1879	Brachyura	2	1	[31]
*Portunus sanguinolentus* (Herbst, 1783)	Brachyura	2	1	[60]
*Scylla serrata* (Forskål, 1775)	Brachyura	2	1	[15]
*Spiralothelphusa hydrodroma* (Herbst, 1794)	Brachyura	2	1	[12]
*Crangon crangon* (Linnaeus, 1758)	Caridea	0	0	[31]
*Neocaridina davidi* (Bouvier, 1904)	Caridea	0	0	[61]
*Palaemonetes argentinus* Nobili, 1901	Caridea	0	0	[62]
*Penaeus bennettae* R. & D., 1965	Dendrobranchiata	2	1	[63]
*Penaeus setiferus* (Linnaeus, 1767)	Dendrobranchiata	1	1	[64]
*Penaeus vannamei* (Boone, 1931)	Dendrobranchiata	1	1	[65]
*Jaxea nocturna* Nardo, 1847	Gebiidea	0	1	[57]
*Upogebia pusilla* (Petagna, 1792)	Gebiidea	0	1	[57]
*Armadillidium arnasus* Budde-Lund, 1885	Isopoda	0	0	[66]
*Armadillidium vulgare* (Latreille, 1804)	Isopoda	0	0	[66]

### Origin of the midgut caeca

The results obtained in this study indicate that the midgut caeca of *M. brachydactyla* are organs originated from the endoderm germ layer. This origin is supported by the anatomical position of the midgut caeca, as well by the light and electron microscopy observations. In crustacean decapods, the position of the midgut caeca in relationship with other digestive organs has been historically a controversial topic (Table 2). The careful dissection of *M. brachydactyla* showed that the midgut caeca derivate from the midgut tract in all the life stages. The same was observed in the micro-CT reconstructions and the light microscopy sections of the larval stages. Since the midgut caeca arise from the midgut tract it is reasonable to propose that those organs have the same germ layer origin: the endoderm germ layer [24, 25, 27].

**Table 2:** Historical summary of the midgut caeca: denomination, derivation, and potential role for the anterior and posterior midut caeca in different decapod taxa. The proposed digestive organ from which the midgut caeca derivate is indicated: HGT, hindgut tract; MGT, midgut tract; MHJ, midgut-hindgut junction; STO, stomach. N.A. “Species” indicates that several species were included in the study and results were general for higher taxa. N.A. in remaining columns indicates that such topic was not available in the referenced study

Species	Taxa	anterior midgut caeca		posterior midgut caeca/caecum		Potential role	Ref.
		denomination	origin	denomination	origin		
N.A.	Brachyura	coecums pyloriques	STO	coecums postérieurs	MHJ	secretion	[11, 67]
N.A.	Brachyura	long membranous appendages	MG	N.A.	N.A.	N.A.	[68]
*Astacus astacus* (Linnaeus, 1758)	Astacidea	caecum	MG	N.A.	N.A.	N.A.	[69]
*Nephrops norvegicus* (Linnaeus, 1758)	Astacidea	dorsal caecum	MG	dorsal diverticulum	MG	absorption inconclusive	[36]
*Pagurus bernhardus* (Linnaeus, 1758)	Anomura	anterior caeca	MG	posterior caecum	MG	inconclusive data	[55]
*Spiralothelphusa hydrodroma* (Herbst, 1794)	Brachyura	midgut caeca	MG	hindgut caecum	HG	absorption	[12]
N.A.	Brachyura	N.A.	N.A.	posterior tubular gland	MG or HG	secretion inconclusive	[70]
N.A.	Brachyura	N.A.	N.A.	posterior diverticulum	HG	osmoregulation	[71]
*Metapenaeus bennettae* Racek & Dall, 1965	Dendrobranchiata	anterior diverticula	MG	posterior diverticulum	MG	secretion and osmoregulation	[63]
*Penaeus chinensis* (Osbeck, 1765)	Dendrobranchiata	intestinal caecum	MG	N.A.	N.A.	secretion	[72]
*Homarus gammarus* (Linnaeus, 1758)	Astacidea	anterior diverticulum	MG	posterior diverticulum	MG	intracellular digestion	[15, 56]
*Pachygrapsus crassipes* Randall, 1840	Brachyura	N.A.	N.A.	posterior midgut caecum	MG	inconclusive	[73]
N.A.	Brachyura	anterior midgut caeca	MG	posterior midgut caecum	MG	diverse functions	[31]
*Metacarcinus magister* Dana, 1852	Brachyura	anterior midgut caeca	MG	posterior midgut caecum	MG	osmoregulation	[59]
*Metacarcinus magister* Dana, 1852	Brachyura	anterior midgut caeca	MG	posterior midgut caecum	MG	digestive processes	[37]
*Menippe rumphii* (Fabricius, 1798)	Brachyura	midgut caeca	MG	hindgut caecum	MHJ	N.A.	[58]
*Portunus sanguinolentus* (Herbst, 1783)	Brachyura	midgut caeca	MG	hindgut caecum	MHJ	digestion & absorption	[60]
*Penaeus setiferus* (Linnaeus, 1767)	Dendrobranchiata	anterior midgut diverticulum	MG	posterior midgut diverticulum	MG	inconclusive	[33, 64]
*Paralithodes camtschaticus* (Tilesius, 1815)	Anomura	anterior midgut caeca	MG	posterior midgut caecum	MG	nutrient absorption inconclusive	[32]
*Maja brachydactyla* Balss, 1922	Brachyura	anterior midgut caeca	MG	posterior midgut caecum	MG	active secretion observed	Present study

Nevertheless, the organ derivation is not enough to identify the germ layer that originates an organ because ectoderm and endoderm derived organs can be continuous between them, e.g. stomach and midgut tract, and midgut tract and hindgut tract [19, 25, 27, 28]. This question can be solved studying the features of the epithelium. In crustaceans, the digestive organs derived from the ectoderm germ layer are the esophagus, stomach, and the hindgut tract (including the anus), and all those organs have an epithelium covered by a cuticle lining [24, 25]. Moreover, the TEM observations reveal an special cell type called “tendon cells”, characterized by dense packs of filaments crossing the entire cell height to connect the upper cuticle with the underlying muscle fibres [48, 51, 74]. In *M. brachydactyla*, both cuticle lining and packs of filaments were identified in the epithelia of the esophagus, stomach, and hindgut tract [28, 48, 50, 51], but neither the cuticle lining or the packs of filaments were found in the epithelial cells of the midgut caeca as presented in this study.

On the other hand, the digestive organs derived from the endoderm germ layer are the midgut tract and the midgut gland, and are characterized by a brush border in direct contact with the lumen and a high secretory activity [24, 25]. Thus, the electron microscopy reveals that those epithelia contain cells with the features of the secretory cells: abundance of mitochondria, rough endoplasmic reticulum, Golgi complexes and ribosomes [18, 25, 27]. Previous studies used all those features to propose that the midgut caeca derivate from the endoderm [15, 70]. In *M. brachydactyla*, all those features were reported previously in both midgut tract and midgut gland [20, 28, 75], and were also identified in the epithelial cells from the midgut caeca during the realization of this study. Hence, the features of the epithelia confirm the midgut caeca as endoderm derived organs.

### Development of the midgut caeca

The midgut caeca of *M*. *brachydactyla* appear as small buds in the newly hatched larvae, enlarge linearly during the larval development, and then continue growing until became elongated and coiled blind-tubules in the adult phase. The earlier references of the midgut caeca in the larval stages of brachyuran crabs include the work of Gerbe [68], which simply reported the AC as “small ampullae” that “become the long membranous appendages” of the adults during development; and Schlegel [76], that identified the PC as a “caecum” in histological sections from the first zoeal stage. Few studies have been published afterwards, K Nakamura [35] identified a pair of AC in the zoeal stages of the gazami crab *Portunus trituberculatus* (Miers, 1876); and Jantrarotai and Sawanyatiputi [34] did the same regarding the zoeal stages of the mud crab *Scylla olivacea* (Herbst, 1796). Pugh [70] reported that the length of the PC increased during the larval development in the fiddler crabs of the genus *Uca*. More recently, Spitzner et al. [19] published a study focused on the organogenesis of the European shore crab *Carcinus maenas* (Linnaeus, 1758) showing the AC and PC through micro-CT reconstructions of different larval stages, and as observed in this study, the midgut caeca of *C. maenas* elongate during the larval development.

The development of the midgut caeca in brachyurans (as *M. brachydactyla* or *C. maenas*) consists on a general lengthening similar to the described in anomurans [32]. The similar development and number of midgut caeca observed in anomurans and brachyurans is coherent with their sister group relationship as Meiura [2, 3]. In contrast, in other decapods the development of the midgut caeca varies considerably. The American lobster *Homarus americanus* H. Milne-Edwards, 1837 has a pair of AC at hatching that degenerate and fuse into a single short caecum [29, 30], as reported in the prawns *Penaeus setiferus* (Linnaeus, 1767) [33] and *Penaeus vannamei* Boone, 1931 [65]. Regarding the PC, in the American lobster its enlarges during development [29, 30], but in penaeid prawns the PC develop into a complex cauliflower-shaped organ [33, 65, 77]. Altogether, this information suggests that the pathways of development of the midgut caeca are highly dependent of the morphology of those organs in the adult stage, and consequently should be related with the phylogeny of the group.

The comparison of the epithelial cells between the larval and adult stages showed a similar organization regarding the type and location of the cellular organelles, but in adults the epithelial cells are taller and the organelles related with the secretory activity are much more abundant (ribosomes, mitochondria, rough endoplasmic reticulum, and Golgi complexes). Similarly, in the midgut gland of *M. brachydactyla* the adult epithelial cells are also taller and richer in organelles than the larval ones [20]. The increased number of those organelles in the adult epithelial cells can be associated with an increased secretory activity, as will be discussed later. The electron-dense tubular structures of the adult epithelial cells might be homologous to the basal membrane folds (extended in tubular structures) found on the larvae. Similar tubular structures reported in the digestive epithelium of the decapods have been interpreted as smooth endoplasmic reticulum [20, 25, 78], albeit other studies recognize these tubules as cell membrane infolds [73, 79, 80]. Other changes observed on the midgut caeca of *M. brachydactyla* were found on the muscle fibres and connective, which were thicker in the adults than in the larvae (in which electron microscopy is required to be identified). The thickening of the different tissue layers during development could be related with the increment of the size of the organ, i.e. a larger organ will requires larger musculature for their motility and larger connective for their sustainability [51].

### Potential role of the midgut caeca

The role of the midgut caeca has been historically one of the major unsolved questions on these organs. Different roles have been suggested: secretion, absorption, intracellular digestion, and even osmoregulation (Table 2). The electron microscopy observations (TEM and SEM) realized in this study showed that the AC and PC of the brachyuran *M*. *brachydactyla* are active organs with an important secretory activity, at least during the adult phase. Curiously, almost two centuries ago H Milne-Edwards [67] was one of the first authors to propose a secretory role for the midgut caeca.

The midgut caeca of *M. brachydactyla* did not show clear evidence of secretory activity during the larval stages, if present, such secretory activity should be marginal. On the contrary, macroapocrine and microapocrine were the secretory mechanisms observed in the midgut caeca of adult *M*. *brachydactyla*. The macroapocrine secretion implies that the cell releases a fragment of cytoplasm into the lumen, this process often involves apical protrusions [81, 82]. Little information is available in the literature regarding the secretory activity of the midgut caeca of the decapods. Pugh [70] only observed merocrine secretion in the PC of the fiddler crabs, a type of secretory activity rarely observed in this study. On the other hand, our observations coincided with Dall [63], which reported vesicle formation and extrusion in the midgut caeca of the prawn *Metapenaeus bennettae* Racek & Dall, 1965, and proposed that the caeca should be very active secretory organs. Moreover, Pinn et al. [57] published SEM pictures of the brush border of the PC of different mud and ghost shrimp species (Axiidea and Gebiidea), showing numerous “coccoid bodies” that resembled the vesicles of secretion observed in this study. The apocrine secretion has been proposed as a mechanism to provide in mass delivery of complex mixtures of peptides and proteins [81].

The microapocrine secretory activity observed in the midgut caeca of the adult *M*. *brachydactyla* was particularly interesting; and it consists on the extrusion of small vesicles from the brush border microvilli. To our knowledge, only two studies reported this type of secretion in the digestive system of the decapods. Castejón et al. [20] observed this kind of secretion in the B-cells of the midgut gland of *M. brachydactyla*. Moreover, Sonakowska et al. [61] reported “small bulges of microvilli” on the epithelial cells from the midgut tract of the freshwater shrimp *Neocaridina davidi* (Bouvier, 1904) (previously named *N. heteropoda*), but in our opinion the published pictures were unclear resolving these bulges as secretory activity or as undulated microvilli. On the other hand, the extrusion of small apocrine vesicles from the microvilli has been widely observed in the midgut tract of the insects, receiving the name of “microapocrine mechanism” or “microapocrine route” [83–86]. The microapocrine vesicles produced by the midgut tract of insects larvae contain high diversity of digestive enzymes, including amylase, lipase, and peptidases, being suggested that these enzymes attach to the peritrophic membrane [83, 85, 87].

The high macroapocrine and microapocrine secretory activity observed in the midgut caeca of *M. brachydactyla* is congruent with the detection of two types of enzymatic activities, amylolytic and proteolytic, in the fluids of the midgut caeca of the Dungeness crab *Metacarcinus magister* (Dana, 1852) by Holliday et al. [37]. Moreover, McGaw and Reiber [88] studying the passage of the food through the digestive system of the blue crab *Callinectes sapidus* Rathbun, 1896 reported that the food remains in the midgut during at least 4 h before entering the hindgut tract. Altogether, we propose that the secretions from the midgut caeca mix with the processed food that enters into the midgut tract, participating in the digestive processes. The anterior location of the AC seems logical according to this hypothesis, allowing the caeca fluids to mix with the food as soon as it enters into the midgut tract. The midgut gland is another major digestive organ involved in the digestion and nutrient absorption connected anteriorly with the midgut tract [18, 20, 27], so the secretions produced by the AC also could enter in the midgut gland participating in the digestive processes. The location of the PC, just before the hindgut tract, is harder to explain. The midgut tract content and the secretions of the PC still could be mixed during the hours in which the food remains inside the midgut tract [88]. Alternatively, the peritrophic membrane must be considered. The peritrophic membrane is secreted by the midgut tract epithelium as a chitin and proteinaceous matrix that surrounds all the digestive content that enter into the hindgut tract [18, 24, 89]. Different studies detected proteins related with digestive and immunological functions immobilized in the matrix of the peritrophic membrane of insects [90, 91], and decapods [92]. So, it is congruent to propose that enzymes and other potential proteins synthetized by the PC could end attached to the peritrophic membrane.

Considering the results obtained in this study, it is expected that the secretory activity of the midgut caeca of *M. brachydactyla* increases as the animal develops and grows, without discarding ontogenetic changes in the digestive enzymatic capacities [93]. The midgut caeca of the adults are enlarged versions of the those of the larvae, showing epithelial cells with taller size and richer in organelles, and a secretory activity increased as well. In *M. brachydactyla*, the comparison between larval and adult versions of other digestive organs also showed differences more related with a larger body size rather than with a true functional change [20, 48, 51], maybe with the single exception of the stomach [49]. This proposal contrasts with studies realized in other decapods. The larvae of the *H. americanus* have a pair of AC involved in the yolk digestion and absorption that degenerate shortly after the yolk depletion [29, 30]; while in the penaeid prawns has been suggested that the AC realize a function equivalent than the midgut gland while this organ remains undeveloped, ceasing such function as the midgut gland develops during the juvenile growth [64, 94]. The different development of the midgut caeca of the brachyuran crabs in comparison to other decapods, probably responds to the divergent evolution and role of the midgut caeca in each decapod taxa.

Alternative functions were proposed for the midgut caeca (Table 2). The midgut caeca could participate in the production of the peritrophic membrane. However, our TEM observations of the lumen of the midgut caeca did not found the layered matrix that could permit to identify the peritrophic membrane [92, 95, 96], and Holliday et al. [37] demonstrated that the ligation of the AC did not affects the production of the peritrophic membrane. Other hypothesis suggests that the midgut caeca could be involved in the absorption and storage of nutrients, but none of such activities has been reported in this study, and feeding experiments realized on decapods never found particulate material in the lumen of the midgut caeca [31, 63]. Finally, the midgut caeca could be related with osmoregulatory processes. This study reported abundant tubular structures and mitochondria in the basis of the epithelial cells, a feature typically associated with osmoregulatory capacities [59, 97, 98]. However, an osmoregulatory role is not supported by other observations, i.e. experiments realized in other brachyuran crabs did not showed a clear response of the midgut caeca to the osmotic stress [37, 73]; and *M. brachydactyla* is a brachyuran species with almost none osmoregulatory capacity and a limited tolerance to osmotic stress [44, 99], so midgut caeca with an osmoregulatory role have not sense in this species. Altogether, little evidence is available to support all these alternative functions for the midgut caeca of the brachyurans.

## Conclusion

This study showed that the anterior and posterior midgut caeca develop from the endoderm germ layer, and thus should be considered another set or organs related with the midgut in decapods. In the common spider crab, the midgut caeca enlarge during development, becoming long and coiled tubules in adults, which have an inner folded surface. This developmental pathway focused on a general surface increment implies that the midgut caeca should have an important role. Electron microscopy revealed an important secretory activity based on macroapocrine and microapocrine mechanisms. Future studies focused on the physiology of the digestion in brachyurans and other decapod taxa should consider the potential implications of the midgut caeca secretions.

## Methods

The local supplier Cademar S. Coop. R. L. (Alcanar, Tarragona) provided the first batch of adult specimens in April 2014. The adults were transported to the Institut de Recerca i Tecnologia Agroalimentàries (IRTA; Sant Carles de la Ràpita, Tarragona, Spain) and kept in 2000 L tanks with the purpose to obtain the larvae. Adults were identified in basis of its North Atlantic origin, large body size, and shape of the anterorbital spine [38, 39, 42, 43, 100]. Lectotype of the species (one adult female) is located at “Spain, Tenerife, Puerto Orotava, 1903, ZSM 603/1” [100]. Voucher larvae of the species (megalopae) are located at “Spain, Barcelona, Institut de Ciències del Mar (ICM) - CSIC, Puerto Orotava, 2006, ICMD002391”. The adults were maintained using the conditions standardized for this species [44]: animal density one male per five-six females in each tank, water renewal rate of 3.5 m^3^ h^− 1^, and natural temperature (18 ± 1 °C) and salinity (35 ± 1 psu), with an additional air supply and fed with fresh and frozen mussels (*Mytilus* sp.). Ovigerous females were not captured. Copulation was observed in captivity, but cannot be discarded fertilization from sperm stored in the seminal receptacles [101]. The larval spawning occurred spontaneously and larvae were recovered 12 h after hatching in designed collectors.

The larvae were cultured to obtain samples to describe the midgut caeca during the larval development. For this purpose, the larvae were cultured in glass beakers (600 ml) at an initial density of 30 larvae per beaker. The beakers were placed inside 360 L tanks used as incubation chambers maintaining constant temperature (21 ± 1 °C) and salinity (35 ± 1 psu). In total were employed 24 beakers. The larvae were fed with *Artemia* sp. (INVE Aquaculture Nutrition, Salt Lake UT, USA) nauplii 24 h post-hatching. Daily, the larvae were pipetted to beakers with clean water and fresh food. The larvae completed the larval development without the requirement of special substrates [45].

### Sampling and gross morphology

Six additional adult specimens were acquired from the supplier and sedated in cold (0–4 °C), a method recommended for different invertebrates [102]. For this purpose, the specimens were kept inside a bucket filled with ice and placed in a fridge, until the adults stop moving before dissection. The dorsal carapace was removed using pliers avoiding to damage the inner organs. The midgut gland (a.k.a. hepatopancreas) was removed carefully to expose the midgut tract. Then, the midgut tract and the anterior caeca (AC) were carefully detached from the stomach, while the hindgut tract was cut after the identification of the posterior caecum (PC). The dissected midgut pieces with the attached caeca were used as follows: two pieces were fixed in 4% formaldehyde for posterior examination, two pieces were fixed for light microscopy (see “Light microscopical analysis” section), and the remaining two pieces were fixed for electron microscopy (see “Electron microscopical analyses (TEM and SEM)” section).

The larvae and first juveniles were sampled daily from the day 0 to the day 12 post-hatching. For this purpose, two beakers per day were selected and removed from the tanks. The larvae from these beakers were gathered and fixed as a whole for the different techniques, being selected from each beaker: 10–20 specimens fixed in 4% formaldehyde to study the midgut caeca development, 4–6 specimens fixed for light light microscopy (see “Light microscopical analysis” section). On day 6 post-hatching, eight additional specimens were selected for Micro-CT (see “Micro-computed tomography” section) and electron microscopy (see “Electron microscopical analyses (TEM and SEM)” section), and on 10 day post-hatching, four megalopae were sampled for electron microscopy. The numbers for sampled larvae varied according to their survival and availability during the culture.

The development of the midgut caeca was studied dissecting the larvae fixed in 4% formaldehyde. The larvae were dissected using dissecting needles and a dissecting microscope. The dissections were not easy, so between 3 and 8 specimens from each day of development were dissected successfully while maintaining the midgut tract and associated caeca intact (average 5.5 ± 1.3 specimens per day during 12 days, including day 0). The image analysis software AnalySIS® (Soft Imaging System, Münster, Germany) was used to photograph and measure the length of each midgut caeca in a total number of 72 specimens. The length variation during the development was analyzed using a general linear model employing R software version 3.6.3 [103]. None length measure was discarded from the analysis.

### Micro-computed tomography

The larval stage in which the midgut caeca were more developed was the megalopa. Then, this stage was selected to be examined using micro-computed tomography (Micro-CT) in the Department of Zoology of the University of Granada (Spain). The micro-CT uses a three dimensional reconstruction of the selected specimen to explore digitally its inner anatomy, without requiring techniques such as dissection or histology [28]. The megalopae were fixed in 70% ethanol and conserved in 100% isopropanol. At continuation, the megalopae were processed for micro-CT by its immersion in a solution of 1% iodine in absolute ethanol during 72 h, followed by hexamethyldisilazane for 4 h, and air-dried overnight. Then, the megalopae were mounted in a piece of Basotect®, a material easy to remove digitally [104, 105]. Scans were performed using a SkyScan 1172 high resolution microtomographer (Bruker microCT; former Skyscan; Kontich, Belgium), a Hamamatsu 80/250 source, and a VDS 1.3Mp camera. The scanning parameters were: isotropic voxel size of 1.47 μm *per* pixel, 54 kV, 85 μA, 0.5° rotation step, and 180° rotation scan. The primary reconstructions and “cleaning” processes used the latest versions of the Bruker microCT software (NRecon, DataViewer, CTAnalyser). CTvox (Bruker’ micro-CT’s Skyscan software) was used to obtain rendered images (Fig. 1: I-K) and Supplementary video 1. For a more detailed description of the procedures, see J Alba-Tercedor [106].

### Light microscopical analysis

The larvae (zoea I, zoea II, and megalopae) were fixed as a whole. In adults, the midgut caeca (AC and PC) were fixed either as a whole or in pieces. The fixative agent was modified Davidson’s fixative (ethanol absolute: seawater: 37% formaldehyde: glycerol: glacial acetic acid in proportion 3: 3: 2: 1: 1). Fixation lasted 24 h, and samples were preserved in 70% ethanol. The samples were dehydrated and infiltrated in paraffin using an automatic tissue processor (Especialidades Médicas Myr, Tarragona, Spain), and blocks were realized using a paraffin processor (Especialidades Médicas Myr, Tarragona, Spain). A Leica RM2155 microtome (Leica Biosystems, Wetzlar, Germany) was used to obtain 2-μm sections. Three staining methods were applied: 1) Haematoxylin-Eosin (HE) to show the general structure; 2) Periodic acid–Schiff (PAS) and Alcian Blue contrasted with Hematoxylin to reveal different polysaccharides and any potential cuticle lining; and 3) Mallory’s trichrome stain (Acid Fuchsine, Orange G and Aniline Blue stains) to highlight the muscular and connective tissues. The stained sections were observed using a light microscope (Leica LB30T 111/97), connected to a camera (Olympus DP70), and the corresponding image analysis software DP Controller version 2.1.1.83 and DP Manager version 2.1.1.163 (Olympus Corporation, Germany).

### Electron microscopical analyses (TEM and SEM)

The TEM observations required the fixation of entire larval specimens (zoeae II and megalopae), and small pieces of the adult midgut caeca (AC and PC). The SEM observations only used pieces of the adult midgut caeca. A solution of 2% paraformaldehyde and 2.5% glutaraldehyde in a cacodylate buffer (0.1 mol L^− 1^ pH 7.4) was used as fixative agent for all the electron microscopy samples. Fixation was realized in constant darkness lasting 12 h at 4 °C. Then, samples were washed twice with cacodylate buffer (0.1 mol L^− 1^, pH 7.4), and post-fixed in 1% osmium tetroxide. Dehydration was realized in increasing series of acetone. The TEM samples were embedded in Spurr’s resin, and a Leica UCT ultra-microtome was used to obtain the semithin and ultrathin sections (60 nm), the latter were stained with uranyl acetate and lead citrate. The TEM observations used a JEOL EM-1010 transmission electron microscope (tungsten filament, 80 kV). The SEM samples were dried using the critical-point drying method, before to be mounted on SEM stubs using self-adhesive carbon stickers. Then, the SEM samples were covered with a carbon coating. The SEM observations used a JEOL JSM-7001F scanning electron microscope (15 kV). The TEM and SEM post-fixative treatments and observations were realized at Centres Científics i Tecnològics de la Universitat de Barcelona (CCiTUB; Hospital Clinic, University of Barcelona, Barcelona).

## Supplementary Information

**Additional file 1.** : Supl. video 1. Micro-CT animated volume-rendered images of 6 days post-hatching megalopa larva, enhancing the midgut tract and associated caeca.

## Data Availability

Data are available from the corresponding author upon reasonable request.

## Abbreviations

AC: Anterior midgut caeca
BF: Basal fold
BL: Basal lamina
CE: Caecum epithelium
CS: Cardiac stomach
CT: Connective tissue
EF: Epithelial fold
EV: Electron-dense vesicle (only in cytoplasm)
FV: Fusion between vesicle (cytoplasm) and apical cell membrane
GB: Golgi body
HE: Hindgut tract epithelium
HGT: Hindgut tract
HV: “Hole” left by a secretory vesicle
LV: Lucent vesicle (only in cytoplasm)
MB: Multivesicular body
ME: Midgut tract epithelium
MF: Muscle fibres
MGG: Midgut gland (a.k.a. hepatopancreas)
MGT: Midgut tract
ML: Multilamellar body
Mt: Mitochondria
Mv: Microvilli
My: Myofibrils
N: Nucleous
PC: Posterior midgut caecum
PS: Pyloric stomach
RER: Rough endoplasmic reticulum
SE: Stomach epithelium
SV: Secretory vesicle
TS: Tubular structures
VL: Vesicle of the lumen
